# Supplementary figures and images for: Salivary molecular spectroscopy: A sustainable, rapid and non-invasive monitoring tool for diabetes mellitus during insulin treatment
Source: PLoS One. 2020 Mar 17;15(3):e0223461. doi: 10.1371/journal.pone.0223461 (PMC7077825; doi:10.1371/journal.pone.0223461)

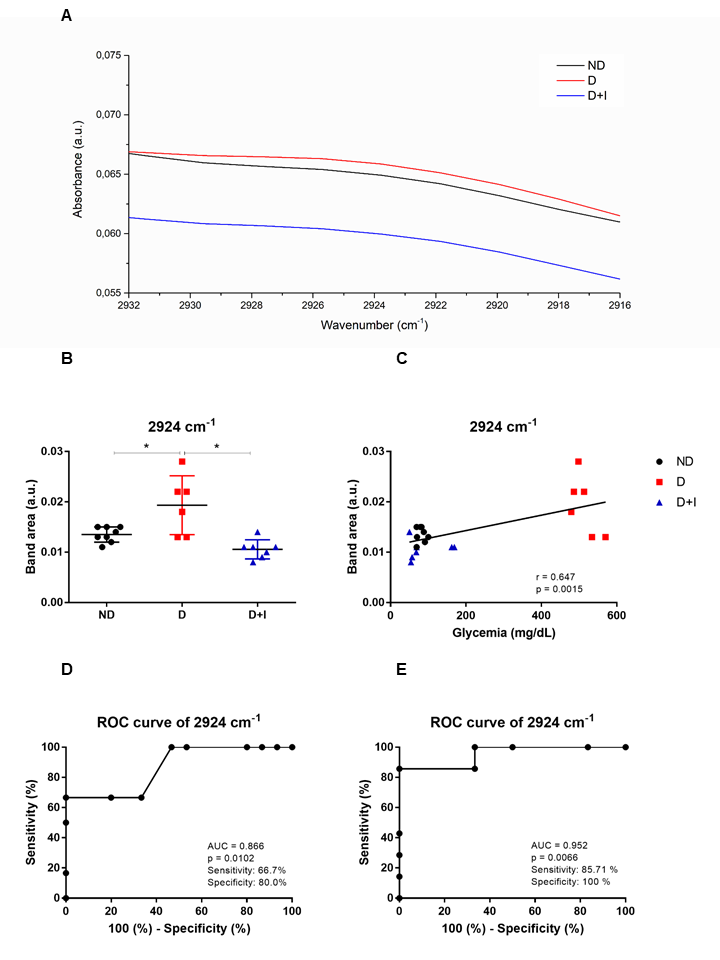

Supplement: S1 Fig — Spectral of 2924 cm-1 (A); Band area of 2924 cm-1 (B); Pearson correlation between glycemia and band area of 2924 cm-1 (C); ROC curve analyses of 2924 cm-1 to normoglycemic and hyperglycemic (D); ROC curve analyses of 2924 cm-1 to diabetic and diabetic treated with insulin (E). Non-diabetic rats (ND), diabetic rats (D) and diabetic treated with insulin (D+I). (TIF) [file pone.0223461.s001.tif]

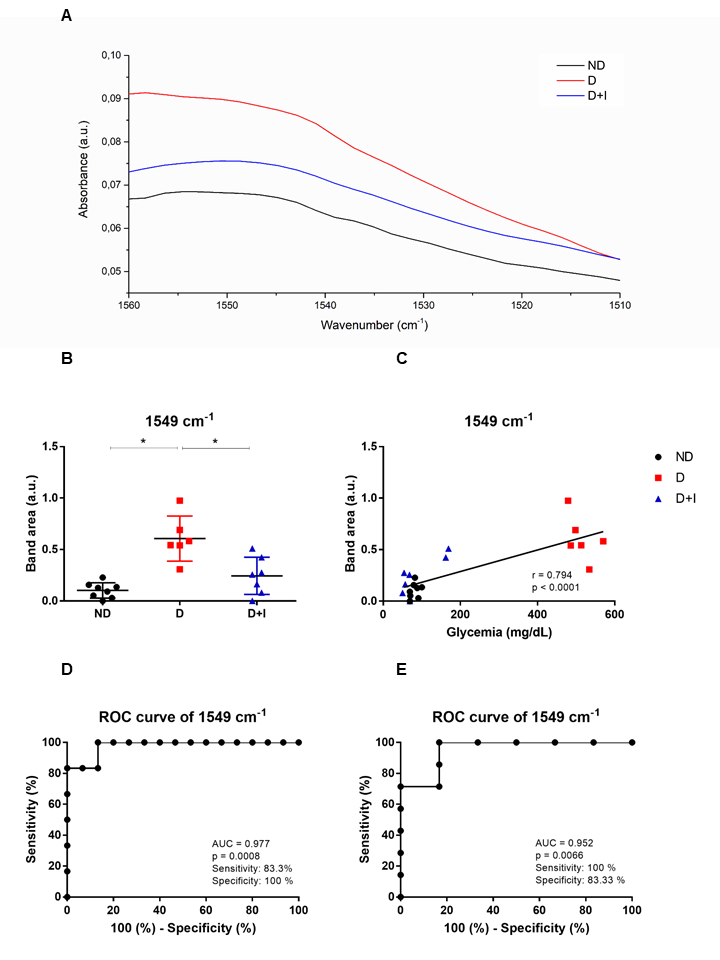

Supplement: S2 Fig — Spectral of 1549 cm-1 (A); Band area of 1549 cm-1 (B); Pearson correlation between glycemia and band area of 1549 cm-1 (C); ROC curve analyses of 1549 cm-1 to normoglycemic and hyperglycemic (D); ROC curve analyses of 1549cm-1 to diabetic and diabetic treated with insulin (E). Non-diabetic rats (ND), diabetic rats (D) and diabetic treated with insulin (D+I). (TIF) [file pone.0223461.s002.tif]

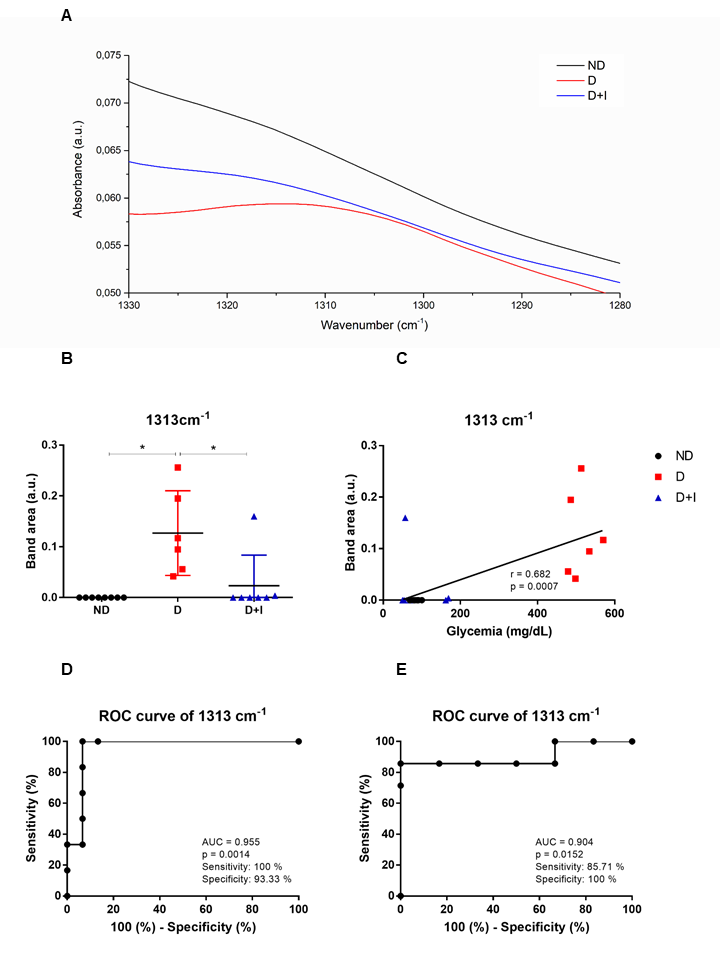

Supplement: S3 Fig — Spectral of 1313 cm-1 (A); Band area of 1313 cm-1 (B); Pearson correlation between glycemia and band area of 1313 cm-1 (C); ROC curve analyses of 1313 cm-1 to normoglycemic and hyperglycemic (D); ROC curve analyses of 1313 cm-1 to diabetic and diabetic treated with insulin (E). Non-diabetic rats (ND), diabetic rats (D) and diabetic treated with insulin (D+I). (TIF) [file pone.0223461.s003.tif]

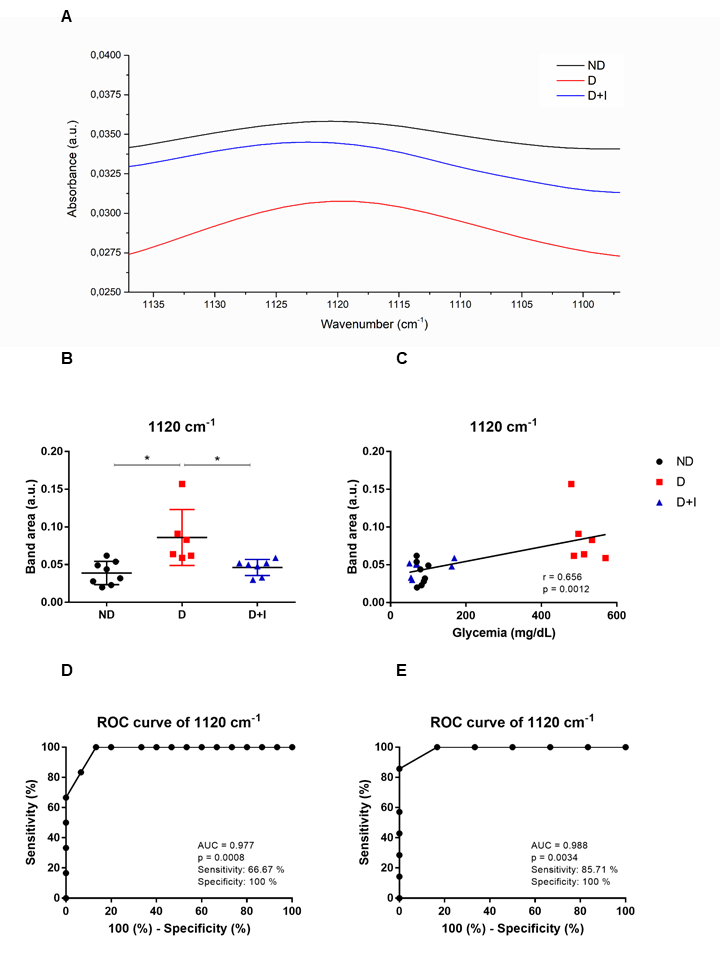

Supplement: S4 Fig — Spectral of 1120 cm-1 (A); Band area of 1120 cm-1 (B); Pearson correlation between glycemia and band area of 1120 cm-1 (C); ROC curve analyses of 1120 cm-1 to normoglycemic and hyperglycemic (D); ROC curve analyses of 1120 cm-1 to diabetic and diabetic treated with insulin (E). Non-diabetic rats (ND), diabetic rats (D) and diabetic treated with insulin (D+I). (TIF) [file pone.0223461.s004.tif]
