# Supplementary material for: Salivary molecular spectroscopy: A sustainable, rapid and non-invasive monitoring tool for diabetes mellitus during insulin treatment
Source: PLoS One. 2020 Mar 17;15(3):e0223461. doi: 10.1371/journal.pone.0223461 (PMC7077825; doi:10.1371/journal.pone.0223461)
Supplement: S1 Table — (DOCX) [file pone.0223461.s005.docx]

**Supplementary Table 1.** Mean quadratic distance in saliva of ND, D and D+I rats.

| Quadratic distance | ND | D | D+I |
| --- | --- | --- | --- |
| D | 0,0000 | 23,3348 | 37,2085 |
| D+I | 23,3348 | 0,0000 | 11,5541 |
| ND | 37,2085 | 11,5541 | 0,0000 |
