# Supplementary material for: Salivary molecular spectroscopy: A sustainable, rapid and non-invasive monitoring tool for diabetes mellitus during insulin treatment
Source: PLoS One. 2020 Mar 17;15(3):e0223461. doi: 10.1371/journal.pone.0223461 (PMC7077825; doi:10.1371/journal.pone.0223461)
Supplement: S2 Table — (DOCX) [file pone.0223461.s006.docx]

**Supplementary Table 2.** Discriminant linear function in saliva of ND, D and D+I rats.

|  | ND | D | D+I |
| --- | --- | --- | --- |
| Constant | -7,105 | -1,663 | -3,374 |
| CP1 | 20,686 | 1,288 | -16,659 |
| CP2 | 34,740 | -8,064 | -19,007 |
| CP3 | 18,897 | -0,100 | -14,095 |
| CP4 | -3,054 | 5,305 | -2,359 |
| CP5 | 4,356 | -9,836 | 5,357 |
| CP6 | 5,835 | -0,779 | -3,699 |
