# Supplementary material for: Salivary molecular spectroscopy: A sustainable, rapid and non-invasive monitoring tool for diabetes mellitus during insulin treatment
Source: PLoS One. 2020 Mar 17;15(3):e0223461. doi: 10.1371/journal.pone.0223461 (PMC7077825; doi:10.1371/journal.pone.0223461)
Supplement: S3 Table — (DOCX) [file pone.0223461.s007.docx]

**Supplementary Table 3.** Summary of classification with the quadratic distance of each sample, prediction, validation and probability of each sample in saliva of ND, D and D+I rats.

|  |  |  |  |  | Quadratic distance | |  | |  |  | |
| --- | --- | --- | --- | --- | --- | --- | --- | --- | --- | --- | --- |
|  | True  group | Predicted.  group | Val-X  Group |  |  |  |  | Probability | | |  |
| Sample |  |  |  | Group | Pred. | Val-X | Predicted | |  | Val-X | |
| 1 | ND | ND | ND | D | 58.073 | 72.570 | 0.00 | |  | 0.00 | |
|  |  |  |  | D+I | 23.160 | 29.507 | 0.00 | |  | 0.00 | |
|  |  |  |  | ND | 6.079 | 12.211 | 1.00 | |  | 1.00 | |
| 2 | ND | ND | ND | D | 36.580 | 34.838 | 0.00 | |  | 0.00 | |
|  |  |  |  | D+I | 11.348 | 11.078 | 0.03 | |  | 0.14 | |
|  |  |  |  | ND | 4.371 | 7.464 | 0.97 | |  | 0.86 | |
| 3 | ND | ND | ND | D | 35.359 | 33.417 | 0.00 | |  | 0.00 | |
|  |  |  |  | D+I | 10.335 | 9.816 | 0.02 | |  | 0.07 | |
|  |  |  |  | ND | 2.961 | 4.497 | 0.98 | |  | 0.93 | |
| 4 | ND | ND | ND | D | 33.837 | 31.958 | 0.00 | |  | 0.00 | |
|  |  |  |  | D+I | 20.628 | 22.550 | 0.00 | |  | 0.00 | |
|  |  |  |  | ND | 3.528 | 5.608 | 1.00 | |  | 1.00 | |
| 5 | ND | ND | ND | D | 63.675 | 88.572 | 0.00 | |  | 0.00 | |
|  |  |  |  | D+I | 34.276 | 54.739 | 0.00 | |  | 0.00 | |
|  |  |  |  | ND | 6.646 | 14.182 | 1.00 | |  | 1.00 | |
| 6 | ND | ND | ND | D | 29.741 | 28.369 | 0.00 | |  | 0.00 | |
|  |  |  |  | D+I | 9.677 | 9.203 | 0.05 | |  | 0.16 | |
|  |  |  |  | ND | 3.678 | 5.918 | 0.95 | |  | 0.84 | |
| 7 | ND | ND | ND | D | 40.529 | 38.586 | 0.00 | |  | 0.00 | |
|  |  |  |  | D+I | 8.646 | 8.220 | 0.02 | |  | 0.03 | |
|  |  |  |  | ND | 1.065 | 1.409 | 0.98 | |  | 0.97 | |
| 8 | ND | ND | ND | D | 31.222 | 29.651 | 0.00 | |  | 0.00 | |
|  |  |  |  | D+I | 5.711 | 5.542 | 0.21 | |  | 0.39 | |
|  |  |  |  | ND | 3.021 | 4.612 | 0.79 | |  | 0.61 | |
| 9 | D | D | D | D | 6.582 | 15.951 | 1.00 | |  | 1.00 | |
|  |  |  |  | D+I | 26.329 | 27.438 | 0.00 | |  | 0.00 | |
|  |  |  |  | ND | 42.640 | 44.320 | 0.00 | |  | 0.00 | |
| 10 | D | D | D | D | 6.150 | 14.176 | 1.00 | |  | 1.00 | |
|  |  |  |  | D+I | 35.688 | 42.839 | 0.00 | |  | 0.00 | |
|  |  |  |  | ND | 41.710 | 42.420 | 0.00 | |  | 0.00 | |
| 11 | D | D | D | D | 4.543 | 8.862 | 1.00 | |  | 1.00 | |
|  |  |  |  | D+I | 24.542 | 23.925 | 0.00 | |  | 0.00 | |
|  |  |  |  | ND | 41.537 | 41.006 | 0.00 | |  | 0.00 | |
| 12 | D | D | D | D | 5.014 | 10.244 | 1.00 | |  | 0.99 | |
|  |  |  |  | D+I | 21.611 | 20.666 | 0.00 | |  | 0.01 | |
|  |  |  |  | ND | 41.594 | 41.372 | 0.00 | |  | 0.00 | |
| 13 | D | D | D | D | 5.526 | 11.899 | 1.00 | |  | 0.92 | |
|  |  |  |  | D+I | 17.901 | 16.907 | 0.00 | |  | 0.08 | |
|  |  |  |  | ND | 28.636 | 27.276 | 0.00 | |  | 0.00 | |
| 14 | D | D | D | D | 9.967 | 40.402 | 1.00 | |  | 1.00 | |
|  |  |  |  | D+I | 51.721 | 117.859 | 0.00 | |  | 0.00 | |
|  |  |  |  | ND | 64.915 | 127.897 | 0.00 | |  | 0.00 | |
| 15 | D+I | D+I | D+I | D | 45.891 | 67.075 | 0.00 | |  | 0.00 | |
|  |  |  |  | D+I | 6.835 | 15.774 | 1.00 | |  | 1.00 | |
|  |  |  |  | ND | 22.101 | 29.173 | 0.00 | |  | 0.00 | |
| 16 | D+I | D+I | D+I | D | 34.031 | 36.870 | 0.00 | |  | 0.00 | |
|  |  |  |  | D+I | 4.261 | 7.567 | 0.88 | |  | 0.54 | |
|  |  |  |  | ND | 8.291 | 7.852 | 0.12 | |  | 0.46 | |
| 17 | D+I | D+I | D+I | D | 31.428 | 32.102 | 0.00 | |  | 0.00 | |
|  |  |  |  | D+I | 3.134 | 5.055 | 0.93 | |  | 0.79 | |
|  |  |  |  | ND | 8.210 | 7.754 | 0.07 | |  | 0.21 | |
| 18 | D+I | D+I | D+I | D | 23.558 | 22.563 | 0.00 | |  | 0.00 | |
|  |  |  |  | D+I | 3.718 | 6.296 | 0.97 | |  | 0.87 | |
|  |  |  |  | ND | 10.479 | 10.037 | 0.03 | |  | 0.13 | |
| 19 | D+I | D+I | D+I | D | 23.534 | 22.983 | 0.00 | |  | 0.00 | |
|  |  |  |  | D+I | 5.454 | 10.844 | 1.00 | |  | 1.00 | |
|  |  |  |  | ND | 24.905 | 31.891 | 0.00 | |  | 0.00 | |
| 20 | D+I | D+I | D+I | D | 29.999 | 30.895 | 0.00 | |  | 0.00 | |
|  |  |  |  | D+I | 4.318 | 7.707 | 1.00 | |  | 1.00 | |
|  |  |  |  | ND | 19.325 | 21.357 | 0.00 | |  | 0.00 | |
| 21 | D+I | D+I | D* | D | 13.77 | 13.15 | 0.21 | |  | 1.00 | |
|  |  |  |  | D+I | 11.15 | 51.72 | 0.79 | |  | 0.00 | |
|  |  |  |  | ND | 26.44 | 62.40 | 0.00 | |  | 0.00 | |
