# Supplementary material for: Salivary molecular spectroscopy: A sustainable, rapid and non-invasive monitoring tool for diabetes mellitus during insulin treatment
Source: PLoS One. 2020 Mar 17;15(3):e0223461. doi: 10.1371/journal.pone.0223461 (PMC7077825; doi:10.1371/journal.pone.0223461)
Supplement: Attachment — Submitted filename: Caixeta et al 2020 Rebutal letter final .docx [file pone.0223461.s008.docx]

To Clemens Fürnsinn, Ph.D.
Academic Editor
PLOS ONE

Thank you for the opportunity to submit a revised version of the manuscript that addresses the points raised during the review process. As you suggested we described some limitations of the present translational platform to be used on the way to the clinic.

We also attached a rebuttal letter that responds to each point raised by reviewers; a marked-up copy of your manuscript that highlights changes made to the original version and an unmarked version of your revised paper without tracked changes.

**To Reviewer #1, #2 and #3**

Comments to the Author

*1. Is the manuscript technically sound, and do the data support the conclusions?*

*The manuscript must describe a technically sound piece of scientific research with data that supports the conclusions. Experiments must have been conducted rigorously, with appropriate controls, replication, and sample sizes. The conclusions must be drawn appropriately based on the data presented.*

*Reviewer #1: Yes*

*Reviewer #2: Partly*

*Reviewer #3: Partly*

**Response: Thank you for your considerations, we believe that our data supports the conclusion of the manuscript based on the data presented. The experiments were conducted rigorously with appropriate controls, replication, and sample sizes based in recent publications of our group in high impact journals (as PMID: 31594931, Nat Commun. 2019; PMID: 31536570, PLoS One. 2019; PMID: 27579154, Oxid Med Cell Longev. 2016; and PMID: 24992094, Nat Protoc. 2014). However, we also agree with several suggestions of the reviewers to improve the manuscript.**

*2. Has the statistical analysis been performed appropriately and rigorously?*

*Reviewer #1: Yes*

*Reviewer #2: I Don't Know*

*Reviewer #3: N/A*

**Response: Thank you for your considerations, the statistical analyses were performed based in statistics guidelines and used in our previous papers (as PMID: 31594931, Nat Commun. 2019; PMID: 31596327; Braz Dent J. 2019; PMID: 31536570, PLoS One. 2019; PMID: 27579154, Oxid Med Cell Longev. 2016; and PMID: 24992094, Nat Protoc. 2014) and other research groups (PMID: 28676642, Sci Rep. 2017). We corrected the legends to clarify the analysis, as requested.**

*3. Have the authors made all data underlying the findings in their manuscript fully available?*

*The PLOS Data policy requires authors to make all data underlying the findings described in their manuscript fully available without restriction, with rare exception (please refer to the Data Availability Statement in the manuscript PDF file). The data should be provided as part of the manuscript or its supporting information, or deposited to a public repository. For example, in addition to summary statistics, the data points behind means, medians and variance measures should be available. If there are restrictions on publicly sharing data—e.g. participant privacy or use of data from a third party—those must be specified.*

*Reviewer #1: Yes*

*Reviewer #2: Yes*

*Reviewer #3: No*

**Response: Thank you for your considerations, the data of the manuscripts are present in 5 figures, 1 table, 4 supplementary figures and 4 supplementary tables. Our data presentation is guided by ours articles described above and other reference papers at salivary diagnosis field in translational studies performed in rodents as: 1)PMID: 25317990, Am J Respir Crit Care Med. 2014 (impact factor: 16.494) and 2) PMID: 23880764, J Biol Chem. 2013 (Impact factor: 4.106). We also accepted suggestions to the reviewer #3 to improve our presentation data.**

*4. Is the manuscript presented in an intelligible fashion and written in standard English?*

*PLOS ONE does not copyedit accepted manuscripts, so the language in submitted articles must be clear, correct, and unambiguous. Any typographical or grammatical errors should be corrected at revision, so please note any specific errors here.*

*Reviewer #1: Yes*

*Reviewer #2: Yes*

*Reviewer #3: No*

**Response: Thank you for your considerations, the PONE-D-19-26190 R1 (revised version) was newly edited by native English co-author from UK and two full-time and part-time co-authors with position in universities of Canada and USA, respectively.**

*To Reviewer #1*

*5. Review Comments to the Author*

*Reviewer #1: This is an interesting paper presenting an analysis of a new and simple IR-based method to distinguish among states of hyperglycemia. The presentation is reasonably clear and the methodology seems sound.*

**Response: We are very thankful for your comments, which have helped us improve our manuscript. As you requested, some results had your displayed revised and some detailed of mathematical analyses using multivariated methods were included.**

*I only have minor comments:*

*1. The description of insulin-treated animals as D6U is unnecessarily confusing. These are insulin-treated, no longer hyperglycemic; the units matter for methods but not for the group descriptor. Suggest these be designated as D+I or some comparably easy and more relevant descriptor.*

**Response: You are right! We understand your concern. As you suggest, the new *description of insulin-treated animals* is D+I in the PONE-D-19-26190 R1 (revised version).**

*2. The actual method for insulin treatment should be augmented to include the type of insulin. Why these particular doses (rather than something based on weight or glycemia for example?)*

**Response: We understand your concern. The doses and the type of insulin were selected based in our previous papers with diabetes and insulin treatment** **(PMID: 19238474, J Membr Biol. 2009; PMID: 21746792 J Endocrinol. 2011; PMID: 24157454, Life Sci. 2013; PMID: 27579154, Oxid Med Cell Longev. 2016; PMID: 28791487, Acta Diabetol. 2017; PMID: 20841505 Am J Physiol Endocrinol Metab. 2010). As requested, we inserted the type of insulin in Material in methods section as “NPH insulin, Biohulin N; Biobrás, MG, Brazil” and inserted appropriate references.**

*3. I don't find the continuous analyses of signal AUC against glucose very compelling. This study is set up to provide large group differences, and these come out as clusters of the data with a pseudo-line relating the variables. The more direct presentation of the comparison is what is done with the box/whisker plots.*

**Response: Our data presentation is guided by our articles described above and other reference papers at salivary diagnosis field in translational studies performed *in vivo* animal models as: 1)PMID: 25317990, Am J Respir Crit Care Med. 2014 (impact factor: 16.494) and 2) PMID: 23880764, J Biol Chem. 2013 (Impact factor: 4.106). This is a standard method of presenting this data. We prefer the data presented in scatter plot than box/whisker plots in the present manuscript as it allows the reader to verify the intensity of each data for all samples. In addition, the reader can also verify the mean ± SD, as in box/whisker plots.**

*4. It is of interest whether these peaks are measuring glucose or a directly related carbohydrate, or something entirely separate. Is that known?*

**Response: We understand your concern. We changed the second version of manuscript to these concepts are clearer to the reader. It is important point out that only glucose concentration in the primary saliva (saliva present in acinar lumen of salivary glands and not secreted in mouth) is closely related to plasma glucose concentration. It is expected that glucose reabsorption promoted by Na+-Glucose Cotransporter SGLT1 in the luminal membrane of ductal cells (PMID: 19238474, J Membr Biol. 2009; PMID: 24499577 Diabetol Metab Syndr. 2013) is the main responsible mechanism to explain the low significant correlation of salivary glucose in diabetic patients [PMID: 19503844; J Biomed Biotechnol. 2009; PMID: 4712136, Am J Physiol. 1973; PMID: 3855982 J Oral Med. 1985]. As expected, the commercialization of saliva glucose biosensors has not been used for diabetes management and new strategies need be developed to measure salivary components that reflects glycemia. The ATR-FTIR device is a platform able to analyze asymmetric CH_3_ bending modes of the methyl groups of proteins at 1452 cm^-1^ and C_2_ conformation of sugars at 836 cm^-1^.**

**Besides, it is important bearing in mind that C_2_ conformation of sugars at 836 cm^-1^ do not reflect a presence of glucose, the aldehyde structure for glucose into a cyclic hemiacetal (glucopyranose) occur in C4-C5 bond brings (PMID: 31386704, Phys Chem Chem Phys. 2019) at 1375 cm^-1^ (PMID: 20726541, J Phys Chem B. 2010) We are able to analyze glucose or a directly related carbohydrate or proteins individually, however, these functional groups can be present in other several types of molecules present in other sugars or in the thousands of proteins within the saliva. The ATR-FTIR spectra of D-(+)-Glucose is represented bellow (https://spectrabase.com/spectrum/EStxK0RpFmt).**

**As with other molecular techniques, FTIR is able to detect functional groups present in several components, which leads to the analysis focus in the intensity of each vibrational modes or in multivariate analysis over a detection of specific type of protein/sugar. When our research focus occurs to specific proteins, we have used several other techniques as Western blotting, Proteomic analysis or electrochemical biosensor coupled with specific antibodies.**

*5. I really like the detailed mathematical analyses using multidimensional methods, adds considerably to the paper.*

**Response: We thank the reviewer for their comments**

*6. It is obvious that this now needs to be further tested in humans with various stages of diabetes, and as a potential marker of actual glucose values. Can a method be developed where a portable device can perform the physical measure and undertake computations to give a useful and actionable output? (I realize this is the 'next' project but it seems the logical extension; perhaps a comment in this paper could be added about challenges of moving this into diagnostic or clinical use).*

**Response: We thank the reviewer for their comments and as such have included the below in the text.**

***“The prospect of identifying spectral biomarkers in saliva open new perspectives for monitoring the severity of the diabetes, and compliance to insulin treatment modalities. Considering the similarity of metabolic mechanism between the diabetic hyperglycemic animals models and diabetic patients, we believe that this salivary ATR-FTIR-based diagnostics could be tested in large samples patients to rapidly and inexpensively monitoring diabetes using saliva samples and even open the possibility for point-of-care assays by portable attenuated total reflectance infrared spectroscopic approaches.“***

*7. The English is overall very good but occasionally there are non-standard English phrases (and some grammatical issues). Suggest this paper be passed by a native English speaker in its final version.*

**Response: Thank you for your considerations, the PONE-D-19-26190 R1 (revised version) was newly edited by native English co-author from UK and two full-time and part-time co-authors with position in universities of Canada and USA, respectively.**

*To Reviewer #2*

*5. Review Comments to the Author*

*Reviewer #2: Have not reviewed yet. Please see my confidential note to the editor.*

**Response: Dear Academic Editor, if necessary, we are able to respond in the future.**

*To Reviewer #3*

*5. Review Comments to the Author*

*Reviewer #3: The authors describe an FTIR-based method to detect spectral changes in the saliva of diabetic mice and compare them with control and treated diabetic mice. The study presents some interesting results which need further biochemical validation, in order to verify that the observed spectral changes are useful for glucose monitoring in the saliva.*

**Response: We are very thankful for your comments, which have helped us improve our manuscript, focusing on the main point, that is, the proposal that ATR-FTIR is able to detect functional groups present in several salivary components, which leads to the analysis focus in the intensity of each vibrational modes or in multivariated analysis over a detection of specific component under biochemical validation. Besides, as you pointed out, the present study may contribute to indirectly evaluate glycemic control in diabetic patients using saliva. Some results and discussion were revised, as required.**

*Major comments.*

*1. The main finding of the authors is the observed spectral decrease at 1452 and 836 cm-1. However, it is not clear what these peaks represent. According to the authors, the 1452 peak corresponds to methyl groups of proteins and the 836 groups to sugars. The authors should provide some kind of evidence on which proteins absorb at 1452 and why they are decreased in the diabetic mice. Furthermore, is the 836 a specific signature of glucose in the saliva? If yes, this peak should be increased in diabetic mice that have higher glucose concentrations in their blood.*

**Response: Firstly, as described in conclusion, it is also important point out the very high discriminatory accuracy of 95.2% to discriminate diabetic rats by the PCA-LDA and HCA multivariate models as a main finding of the manuscript. However, we understand your concern. In the PONE-D-19-26190 we do not emphasizes that the focus of ATR-FTIR analyses is not identified specific components. In the PONE-D-19-26190 R1 (revised version) we inserted one sentence to emphasizes that ATR-FTIR is able to detect functional groups present in several salivary components (as different proteins or different types of sugars), which leads to the analysis focus in the intensity of each vibrational modes or in multivariate analysis over a detection of specific component under biochemical validation.**

**The ATR-FTIR devices is a platform able to analyze asymmetric CH_3_ bending modes of the methyl groups of proteins at 1452 cm^-1^ and C_2_ conformation of sugar at 836 cm^-1^. The reduction in asymmetric CH_3_ bending modes of the methyl groups of proteins at 1452 cm^-1^ can be related with the profile of Salivary Proteome and Peptidome in diabetic conditions using quantitative approach. As expected in metabolic diseases, some salivary proteins increase and other salivary proteins (as albumin, alpha2-macroglobulin-like protein 1; defensins, and deleted in malignant brain tumors 1 protein) reduce your concentration in saliva under diabetic conditions (PMID: 23406527, J Proteome Res. 2013). Besides, it is important point out that sympathetic nerve activation to the salivary glands (SNASG) results in a relatively low flow of saliva rich in protein (PMID: 230224726, Physiol Behav. 2012). We also showed that basal SNASG was decreased (∼50%, P < 0.05) in diabetic condition (PMID: 20841505, Am J Physiol Endocrinol Metab. 2010), which can contribute to explain the present data at 1452 cm^-1^. We are able to analyze glucose or a directly related carbohydrate or proteins individually, however, these functional groups can be present in other several types of molecules present in other sugars as fructose, galactose, lactose, maltose and sucrose. Second derivative spectra of glucose are mainly expressed in 1021, 1014, 1080, and 988 cm^−1^ (PMID: 28409627, Anal Chem. 2017).**

**Besides, the C_2_ conformation of sugars at 836 cm^-1^ do not reflect a presence of glucose, the aldehyde structure for glucose into a cyclic hemiacetal (glucopyranose) occur in C4-C5 bond brings (PMID: 31386704, Phys Chem Chem Phys. 2019) at 1375 cm^-1^ (PMID: 20726541, J Phys Chem B. 2010). The ATR-FTIR spectra of D-(+)-Glucose is represented bellow (https://spectrabase.com/spectrum/EStxK0RpFmt).**

**So, the 836 cm^-1^ is not focused on glucose and it is important bearing in mind the low significant correlation of salivary glucose in diabetic patients [PMID: 19503844; J Biomed Biotechnol. 2009; PMID: 4712136, Am J Physiol. 1973; PMID: 3855982 J Oral Med. 1985].** **As expected, the commercialization of saliva glucose biosensors has not been used for diabetes management and new strategies need be developed to measure salivary components that reflects glycemia. The ATR-FTIR devices is a platform able to analyze asymmetric CH_3_ bending modes of the methyl groups of proteins at 1452 cm^-1^ and C_2_ conformation of sugars at 836 cm^-1^. Thus, it is important bearing in mind that the focus of this paper is to find new specific salivary components for diabetes diagnosis/monitoring. We believe that biochemical validation of each vibrational mode is necessary when the study have a biomarker present in specific component, however, in our case the univariate analysis detect the concentration of the functional groups present in several types of proteins and sugars. The innovation of this study is the potential application to a sustainable, rapid and non-invasive monitoring tool for diabetes mellitus during insulin treatment.**

*2. The changes in the spectra of diabetic mice are subtle and measured in arbitrary units. Therefore, they are not convincing. The authors should show the FTIR spectrum of the diabetic mice after having removed as a background either the spectrum of non-diabetic or insulin-treated mice. Then, they would indicate spectral changes as variations from the respective baseline*

**Response: We understand your concern, we believe that the spectra of samples can be measured and presented in arbitrary units based in recent publications of our group in high impact journals (as PMID: 24992094, Nat Protoc. 2014; PMID: 31594931, Nat Commun. 2019) and other research groups (PMID: 28676642, Sci Rep. 2017). However, we agree with your suggestions to display the spectra of diabetic and insulin-treated diabetic samples using non-diabetic samples as reference. This figure was presented in Figure 1.B**

*3. Would the authors detect the 1452 and 836 peak changes also in human samples from diabetic patients?*

**Response: We expected that the changes occurred in animal models of diabetes are able to occur in diabetic patients. The present manuscript is a translational study and we expect to show the clinical data using our protocol with ultra-low values of saliva (2 μl) under airflow dried during only 2 minutes in a future study.**

*4. The paper would benefit if the authors could e.g. measure the FTIR spectra of increasing concentrations of glucose solutions and compare them with the spectra obtained from the 3 different mice groups.*

**Response: We understand your concern. However, we changed the second version of manuscript to clarify that the focus of the manuscript is not evaluate salivary glucose by ATR-FTIR. The glucose reabsorption promoted by Na+-Glucose cotransporter SGLT1 in the luminal membrane of ductal cells (PMID: 19238474, J Membr Biol. 2009; PMID: 24499577 Diabetol Metab Syndr. 2013) can explain the low significant correlation of salivary glucose in diabetic patients [PMID: 19503844; J Biomed Biotechnol. 2009; PMID: 4712136, Am J Physiol. 1973; PMID: 3855982 J Oral Med. 1985]. As expected, the commercialization of saliva glucose biosensors has not been used for diabetes monitoring and new strategies need be developed to measure other salivary components to reflects glycemia. Second derivative spectra of glucose are expressed in 1031, 1014, 1080, and 988 cm^−1^ (PMID: 28409627, Anal Chem. 2017) and our ATR-FTIR platform coupled with univariate analysis focused in different vibrational modes/** **functional groups, as asymmetric CH_3_ bending modes of the methyl groups of proteins at 1452 cm^-1^ and C_2_ conformation of sugar at 836 cm^-1^. We are able to analyze specific components in different concentrations, however, the spectral functional groups at 1452 cm^-1^ and 836 cm^-1^ can be present in other sugars or in the thousands of proteins within the saliva. We believe that biochemical validation of each vibrational mode is necessary when the study have a biomarker present in specific component, however, in our case the univariate analysis detect the concentration of the functional groups present in several types of proteins and sugars. The innovation of this study is the potential application to a sustainable, rapid and non-invasive monitoring tool for diabetes mellitus during insulin treatment.**

*5. In the discussion section, the authors describe that glucose concentration can be measured in the saliva of diabetic individuals. Therefore, the authors should validate the presence of glucose in their saliva samples and measure its concentration. The biochemical validation of their results is missing.*

**Response: We understand your concern, however, although the salivary glucose concentration is increased in diabetic patients, it was showed a low significant correlation of salivary glucose in diabetic patients [PMID: 19503844; J Biomed Biotechnol. 2009; PMID: 4712136, Am J Physiol. 1973; PMID: 3855982 J Oral Med. 1985]. As expected, the commercialization of saliva glucose biosensors has not been used for diabetes monitoring and new strategies need be developed to measure other salivary components to reflects glycemia. Considering your commentary, we rephase the discussion on this point.**

*Minor comments*

*1. The values for the reduced weight gain in Table 1 are misleading. For example, it shows a -2.7 change with a SD of 11.3. Instead, the authors should consider to show the average body weight and its SD. Also, they should clearly state what the * and # symbols indicate.*

**Response: We understand your concern. It was our fault. The data inserted in Table 1 is mean ± SD, however we did not insert this information in previous version. We also attached information about Δ Body weight (g) calculation (the mean of Δ Body weight (g) in diabetic rats was - 2.7 g with 11.3 of SD. We also inserted details about symbols in the legends.**

***P < 0.05 vs ND, #P < 0.05 vs D; one-way ANOVA followed by Student Newman Keuls post-test.**

*2. Readers that are unfamiliar with ROC curves may not easily understand the rationale of these statistical tests and their downstream analysis. The authors should consider to rephrase lines 172-184. Furthermore, usually ROC curves show a (1-specificity) in their x-axis. Here, the authors show specificity in their ROC curves. Is this correct? Also, does each point in these curves represent a single animal? The equal distance between data points seems unusual.*

**Response: We inserted the *lines 172-184 to explain ROC curves to unfamiliar readers. It is a classical analysis to evaluate the powerful of new diagnostics tests. As you suggested, we inserted data in x-axis, however, on % values the ROC curve is represented by 100(%)-specificity (PMID: 28594948, PLOS One, 2017) The points in these curves do not represent samples, the Prisma software® perform several cut-off to display a specific sensitivity and 100(%)-specificity in ROC curves.***

*3. The last two sentences of the results section (lines 208-209) are the conclusion of the PCA analysis. The authors should move them at the end of the previous paragraph.*

**Response: We understand your concern, however, both Principal Component Analysis followed by linear discriminant analysis (PCA-LDA) and Hierarchical Cluster Analysis (HCA) had the same performance. We rephrase the sentence (lines 208-209) as: “The total accuracy, which is highly important for potential monitoring applications, was also 95.2% (20/21)** **in HCA analysis.”**

*4. The authors should consider to shorten the Discussion section. At its present form, it seems very long compared to the other sections of the paper*

**Response: This discussion presents results into a global context based mainly on evidence-based data to explain in order: diabetic state, potential salivary biomarkers to diabetes, spectral biomarkers derived from univariated and multivariated analysis, potential applications and study limitations.**

*5. Description of the animal groups is misleading. The author should state that they originally induced diabetes in 13 animals and then divided them in the D and D6U groups. The first sentence in Line 343 should move after the selection of mice with hyperglycemia.*

**Response: Thank you for your comments. This has been updated in the manuscript**

*6. The authors should use different colors for the marks (animals of each group) in Figure 2C and all other similar figures. Could they also use similar colors for the marks in parts D-E?*

**Response: We are very thankful for your comment to Pearson’s correlation analysis. That was thoroughly revised in Figure 2C and all other similar figures. As we discussed above, the points in these curves do not represent samples (parts D-E).**

*7. The authors should check the legends of Figure 3, 4, S1-S4. Some words seem to be missing. For example, line 716, ROC curve analysis of 836 cm-1 to diabetic mice and diabetic mice treated with insulin.*

**Response: Thank you for your comments. Figure legends have been thoroughly revised.**

*8. In Figure 4, the authors should state the % proportion of variance of analyzed data in the y-axis.*

**Response: This figure has been updated.**

.
