# Supplementary material for: Salivary molecular spectroscopy: A sustainable, rapid and non-invasive monitoring tool for diabetes mellitus during insulin treatment
Source: PLoS One. 2020 Mar 17;15(3):e0223461. doi: 10.1371/journal.pone.0223461 (PMC7077825; doi:10.1371/journal.pone.0223461)
Supplement: Attachment — Submitted filename: Caixeta et al 2020 r2 Rebutal letter final.docx [file pone.0223461.s009.docx]

To Clemens Fürnsinn, Ph.D.
Academic Editor
PLOS ONE

Thank you for the opportunity to submit a new revised version of the manuscript that addresses the last points raised during the review process. We agree with the limitations that you described about the PONE-D-19-26190R1 to our infrared platform be used in the clinic. Although requiring further confirmation to provide that our platform is suitable to detect glycemic fluctuation (minutes/hour), in summary, these salivary results indicate that ATR-FTIR spectroscopy coupled with univariate or multivariate chemometric analysis has the potential to provide a novel noninvasive approach to diabetes monitoring. Supposing that a similar vibrational mode can discriminate against other conditions than hyperglycemia, we can assume that our multivariate chemometric analysis is suitable in discriminate other diseases.

We also attached a rebuttal letter that responds to each point raised by the reviewer#3; a marked-up copy of your manuscript that highlights changes made to the previous version, and an unmarked version of your revised paper without tracked changes.

**To Reviewer #3**

**We are very thankful for your comments, which have helped us improve our manuscript. Besides, as you pointed out, we also agree the present data support the conclusions, and the statistical analysis was performed appropriately and rigorously.**

*Review Comments to the Author*

*Reviewer #3: I realize that the authors decided not to include any new experimental data, as I would expect, but instead of tried to explain their results in the revised Discussion section better. However, within this lengthy section, I don't see a clear statement on what the.g. on their claim that the 1452 spectrum may correspond to several protein components. Mainly, I don't see a clear report on the limitations of their study, merely the value of their experimental approach.*

**Response: We understand your concern. As we described in the R1 rebuttal letter, the suggested analysis with glucose in the saliva is not closely related to present data. Concerning 1452 cm^-1^ vibrational mode, the L153-154 informed that “asymmetric CH3 bending modes of the methyl groups of proteins (1452 cm-1)". It is a scientific form to indicates the presence of this vibrational mode in several proteins in the biospectroscopy field. Besides, in L319-321, you can see another explanation about this point, "As other molecular techniques, the ATR-FTIR can detect functional groups present in several components…”. This is the standard format to provide this information in previous studies from our and other research groups. As requested, we inserted new sentences in the limitation-of-study topic based on the editor and your comments (L-327-333):**

**“Although requiring further confirmation to provide that our platform is suitable to detect glycemic fluctuation (minutes/hour), the present data indicate that our novel noninvasive approach to diabetes monitoring has the potential to provide discrimination of short-time insulin treatment. Supposing that a similar vibrational mode can discriminate against other conditions than hyperglycemia, we can assume that multivariate chemometric analysis is suitable do discriminate against other diseases.”**

*Also, I have seen several inconsistencies in their rebuttal letter.*

1. *The authors claim that inserted Lines 172-184, but I don't see any changes there*

**Response: We understand your concern, sorry if we not clear. We previously added *Lines 172-184*** *[actually in lines 175-182]* ***in* our original version of the manuscript. We believe that the information about the ROC curve with respective sensitivity and specificity described in Methods (L.423 - 435 and L.457-462) is sufficient to the PLOS One readers. We also provide a reference to ROC curve analysis in the Material and methods section (L-434).**

2. *They also state that the manuscript has been thoroughly checked by native English speakers. However, I found more than one mistake. E.g. "Line 320, it is important emphasizes..., Line 181 To emphasizes our focus...., Line 437, the term DU6 that was deleted is used again.*

**Response: Thank you for your comments; all suggestions were made in the current version of the manuscript.**

3. *Figure 4. The authors included the symbol % bit no numerical value for the proportion of variance that each PC diagram explains.*

**Response:** **The numerical value for the proportion of variance that each PC diagram explains was inserted in the result section as requested (L196-198).**

**“The PC1 to PC6 proportions of the spectra variability in the covariance matrix were, 39.0%, 32.0%, 11.3%, 8.2%, 3.1% and 2.2%, respectively.”**

*My overall impression is that the authors have not carefully prepared their revised manuscript and response to the reviewers. I would encourage them to resubmit a more careful revision of their paper, in which they would also clearly state the limitations and potential obstacles on the way to the clinic.*

**Response: Thank you for your comments. A native English speaker has read-proofing the manuscript. As requested, we also improved the study limitation section.**
